# The effects of Arf4 gene knockdown on Alzheimer's amyloid β deposition: Possible role in cognitive resilience

**DOI:** 10.21203/rs.3.rs-11040037/v1

**Published:** 2026-10-14

**Authors:** Sara Hemmati, Erming Wang, Minghui Wang, Boer Xie, Stephen Salton, Vahram Haroutunian, David Bennett, Peng Xu, Junmin Peng, Sam Gandy, Bin Zhang, Michelle Ehrlich

## Abstract

Background Studying the factors involved in cellular and cognitive resilience is a crucial step toward designing preventive and targeted therapeutic approaches for Alzheimer’s disease (AD). Methods Systematic analysis of the proteomic landscape in the advanced aging populations identified ADP ribosylation factor 4 (ARF4) as one of the key driver genes associated with resilience among the oldest-old (OO) non-demented individuals. To assess the effects *of Arf4* gene knockdown in AD animal models, we generated APP ^KM670/671NL^ x PSEN1 ^Δexon9^ (PSAPP)/ *Arf4* ^+/−^ mice. Results Protein signatures and protein coexpression network modules of the oldest-old (OO) non-demented individuals were identified. Integrative protein network analysis further revealed ARF4 as a top protein driver of OO. Mouse experiments showed *that Arf4* knockdown was associated with increased amyloid plaque burden and enhanced microglial activity. The consequence of *Arf4* knockdown was an alteration in amyloid precursor protein (APP) localization, which led to variations in mature APP levels. *Arf4* knockdown further led to transcriptomic alterations in the mouse brain; most pronounced in pathways associated with immune regulation and microglial homeostasis. Conclusion Based on the exacerbated AD pathological features and transcriptomic alterations observed in *Arf4* knockdown animals, ARF4 appears to play a critical role in AD resilience and may represent a potential prognostic factor worthy of further investigation.

